# Conflict between Intrinsic Leaf Asymmetry and Phyllotaxis in the Resupinate Leaves of *Alstroemeria psittacina*

**DOI:** 10.3389/fpls.2012.00182

**Published:** 2012-08-10

**Authors:** Daniel H. Chitwood, Daniel T. Naylor, Paradee Thammapichai, Axelle C. S. Weeger, Lauren R. Headland, Neelima R. Sinha

**Affiliations:** ^1^Department of Plant Biology, University of California at DavisDavis, CA, USA

**Keywords:** morphometrics, leaves, leaf development, resupination, *Alstroemeria*, left-right asymmetry, leaf, plant development

## Abstract

Spiral phyllotactic patterning is the result of intricate auxin transport relationships in the shoot apical meristem (SAM) that act to place auxin maxima at the future sites of leaf initiation. Inherent to this process is a bias in auxin distribution in leaf primordia, such that increased auxin is found on the descending side of the leaf (toward the older neighbor) compared to the ascending side (toward the younger neighbor), creating phyllotactically dependent leaf asymmetry. Separate from phyllotactic-dependent asymmetry is handedness in plants – that is, genetically encoded, fixed chirality, such as the twining of certain vines and the torsions induced by microtubule mutations. Here, we perform a morphometric analysis on the resupinate leaves of *Alstroemeria psittacina*. Interestingly, the twist in leaves always occurs in a single direction, regardless of the phyllotactic direction of the plant. Because of the resupination, leaves in this species possess an inherent handedness. However, this asymmetry is modulated in a phyllotactic-dependent manner, consistent with the known developmental constraints of phyllotaxis upon leaf morphology. This creates the interesting circumstance in *A. psittacina* that leaves arising from plants with a counter-clockwise phyllotactic direction are (1) more asymmetric, (2) larger, and (3) possess symmetrical shape differences relative to leaves from plants with clockwise phyllotaxis. The mechanism underlying these differences likely involves a developmental delay in clockwise leaves caused by the conflict between the phyllotaxis-dependent asymmetry and asymmetry resulting from resupination. The evolutionary implications of a dimorphic population without a genetic basis for selection to act upon are discussed.

## Introduction

Leaf morphogenesis is modulated by a number of developmental signals acting across numerous axes. Proximal/distal outgrowth begins shortly after the specification of the incipient leaf, whose placement is patterned by complex auxin transport processes (Reinhardt et al., 2003; Smith et al., 2006) and the subsequent down-regulation of KNOX activity and other factors specifying indeterminacy (Sinha et al., 1993; Timmermans et al., 1999; Tsiantis et al., 1999). The adaxial/abaxial axis is patterned by a meristem-derived factor that promotes adaxial cell fates (Sussex, 1951; McConnell et al., 2001; Chitwood et al., 2009), and the juxtaposition of adaxial and abaxial identities is necessary for laminar outgrowth (Eshed et al., 2004), thereby producing the medial-lateral axis of the blade (Nardmann et al., 2004; Shimizu et al., 2009). Extensive knowledge about the molecular mechanisms governing the patterning of the aforementioned axes has accumulated (Husbands et al., 2009), and a comprehensive model for how bilaterally symmetric leaves are produced has emerged.

Nonetheless, one axis – the left/right axis – remains understudied. In species with obvious left-right asymmetries, a clear relationship with phyllotaxy has been observed (Korn, 2006). In plants with spiral phyllotaxy, the relevant axis in question – the ascending/descending axis – is conflated with the left/right axis. The ascending direction of a leaf faces the younger neighboring leaf, whereas the descending side faces the older neighbor (we prefer “ascending/descending” as used in Chitwood et al., 2012a for the clear descriptive definition these terms provide, although “anadromic/catadromic,” “anodic/cathodic,” and “dextrorse/sinistrorse” are alternative nomenclatures; see Macloskie, 1895; Raunkiaer, 1919; Dormer, 1972; Korn, 2006). In clockwise (CL) phyllotaxy (ascending the spiral toward the apex in the direction from older to younger leaves) the right side of the leaf is ascending and the left side descending; the converse is true in counter-clockwise (CC) phyllotaxy. In a variety of species, a number of features, including the bending of the midrib, leaf coiling, smaller axillary buds, and secondary blades occur on the ascending side of leaves (Korn, 2006). Importantly, in species where blade outgrowth is obviously asymmetric, such as in *Aglaonema* and *Calathea*, laminar outgrowth occurs more prolifically on the descending side of the leaf toward the older neighbor.

Species exhibiting bilateral asymmetry are thought to be rare. However, if one considers the context of the ascending and descending sides of a young leaf primordium, the left and right sides occupy very different developmental niches. Not only do the two sides of a leaf primordium differ in their proximities to neighboring primordia of various ages, but they also occupy different environments with respect to auxin transport. The ascending side of the leaf is closer to younger primordia that are more likely to act as auxin sinks, whereas the descending side is closer to older primordia more likely to act as auxin sources. As the patterning of leaflets, serrations, and to some extent laminar outgrowth is determined by auxin transport, one might expect the two sides of a leaf to be morphologically distinct. In fact, upon detailed inspection, superficially bilaterally symmetric leaves, such as in tomato and *Arabidopsis*, do exhibit left-right asymmetry (Chitwood et al., 2012a). In tomato, leaflets and lobes are shifted distally on the descending side of the leaf, and in *Arabidopsis*, excess blade outgrowth occurs on the descending side. These features are consistent with modeling and empirical evidence that excess auxin distribution is present on the descending side of primordia, and that ectopic auxin application is sufficient to recapitulate these features.

However, asymmetry in leaves is not limited to the ascending/descending axis. One of the most interesting developmental anomalies in leaves is resupination. Members of *Bomarea* and *Alstroemeria* of the Alstroemeriaceae exhibit resupinate leaves which twist 180° at the petiole to invert the leaf (Lyshede, 2002; Hofreiter and Lyshede, 2006). A number of other species, from diverse families, also exhibit similarly resupinate leaves (as detailed in Hill, 1939), including *Luzuriaga radicans* (Philesiaceae), *Leptaspis cochleata*, and *Pharus latifolius* (Poaceae), and *Stylidium pilosum* (Stylidiaceae). Pitcher plants, as well, can exhibit resupination (Danser, 1928), and resupination in flowers (especially in pendent racemes and various orchids) is prevalent (Ames, 1938; Hill, 1939). In dorsiventrally flattened resupinated leaves, the relative positioning mesophyll cell types and stomatal densities are often reversed from their normal positions along the adaxial/abaxial axis (Hill, 1939), and this is true for many members of *Bomarea* and *Alstroemeria* as well (Lyshede, 2002; Hofreiter and Lyshede, 2006). The abaxial side, therefore, becomes the functional “top” side of the leaf (which is normally adaxial), and takes on the characteristics normally associated with it. This functionality is exemplified by the ability of some *Bomarea* to reposition their abaxial leaf surfaces toward incident light, leading to either an untwisting of the leaf or a double twist (Hill, 1939). To avoid confusion, we refer to the sides of *Alstroemeria* leaves as “abaxial-top” (ab.-top) and “adaxial-bottom” (ad.-bottom) throughout this study.

**Figure 1:**
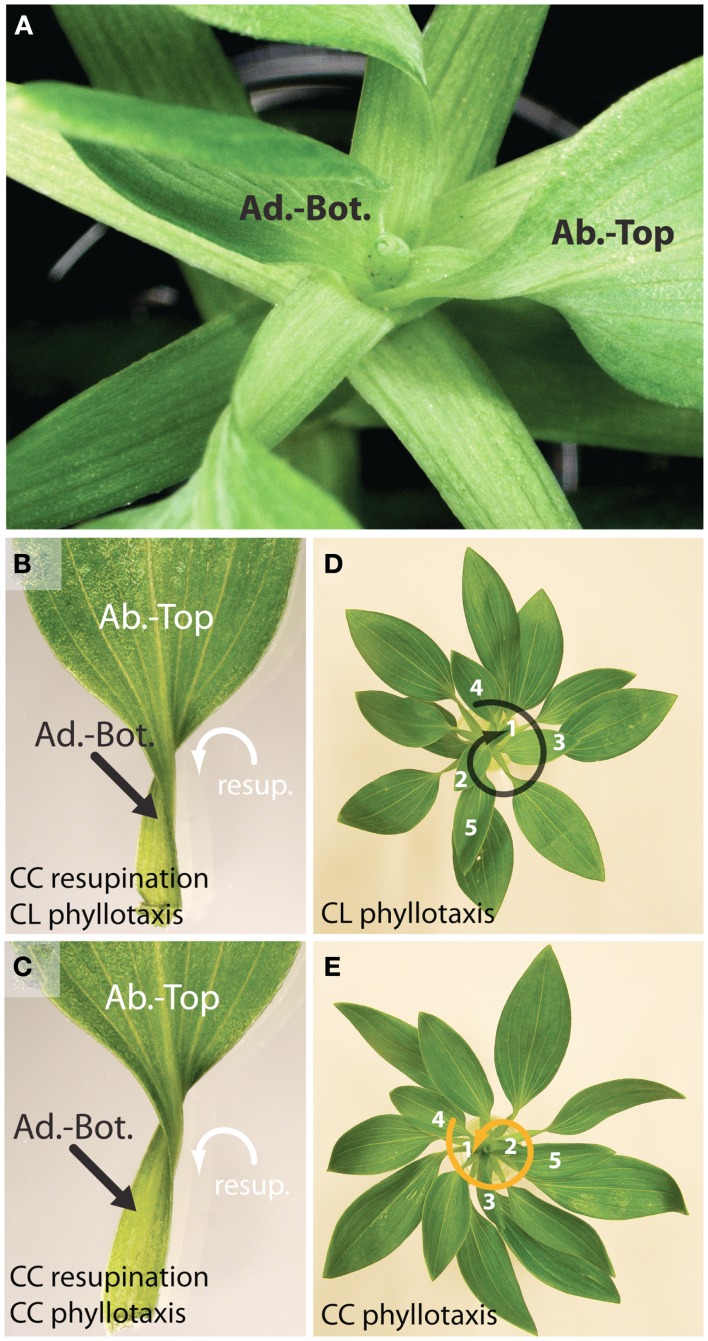
**The direction of resupination is invariant in *A. psittacina***. **(A)** Resupination occurs late in leaf development in *A. psittacina*. Note that the leaf in which the adaxial-bottom side is marked is in the process of inverting. The next leaf in the series, in which the abaxial-top side is marked, has completed its inversion. The leaves that are marked will continue to develop, accentuating the petiole region and growing in size. **(B,C)** Leaves from clockwise [**(B)**, CL] and counter-clockwise [**(C)**, CC] plants, showing that the direction of resupination is invariant. CC turning of the leaf (white arrow) is in respect to the perspective shown, looking down the petiole toward the leaf tip. **(D,E)** Plants exhibiting CL **(D)** and CC **(E)** phyllotaxy. Phyllotactic direction is determined by traversing up the spiral toward the apex, moving from older to younger leaves. As mentioned in the text, there is no statistical deviation in the frequency by which each phyllotactic direction occurs from a 50:50 ratio. Black and orange denote CL and CC phyllotaxis throughout the figures.

Obviously, asymmetric growth must be present to create the resupination seen in the leaves of *Alstroemeria* and other species. In *Alstroemeria psittacina*, leaves initiate with “normal” adaxial-top/abaxial-bottom orientation, and the resupination arises later in leaf development (Figure 1A). Such asymmetric growth could feasibly impart morphological asymmetry in leaves. Interestingly, the numerous leaves we have observed in *A. psittacina* (UC Davis Arboretum, accession number A92.0412) are invariably twisted CC (viewing the leaf from the petiole toward the blade, with the ab.-top side facing upwards, as shown in Figures 1B,C). This consistent CC twist is present whether or not the plants exhibit CL or CC phyllotaxy (Figures 1B–E), and in the shoots from the populations we have examined, *A. psittacina* exhibits no statistically significant bias in the propensity to exhibit phyllotaxy of either direction (assuming 50:50 probability; CL = 100, CC = 109, χ^2^ = 0.388, df = 1, two-tailed *p*-value = 0.5336). The invariable CC twist in *A. psittacina* is distinct from previously described phyllotactic-dependent asymmetries, but reminiscent of other asymmetric phenomena. For example, mutations in microtubule components can induce global asymmetries of a particular handedness, which when manifest in petioles, can approximate the characteristic twist of resupinate leaves (Furutani et al., 2000; Thitamadee et al., 2002). Other examples of handedness that are always of a particular orientation included the twining of vines (Hashimoto, 2002) and examples of fixed phyllotactic direction, such as in *Calathea* (Korn, 2006).

If opposing phyllotactic directions impose opposite leaf asymmetries in *A. psittacina* as they do in other species (Korn, 2006; Chitwood et al., 2012a), and if resupination, which is invariably CC (Figure 1), were to impart asymmetry, what are the effects on the resulting morphology of leaves? Would the asymmetry imparted by resupination depend on the phyllotactic context of the individual? Here, we quantitatively analyze the shape of >2,300 leaves (based on >4,600 images) arising from >240 shoots of *A. psittacina* to determine the sources of asymmetry contributing to leaf shape and their interactions with each other. We determine that the leaves of *Alstroemeria* exhibit a statistically significant intrinsic asymmetry, which we attribute to the invariant direction of resupination. This asymmetry varies by position in the leaf series, but interestingly the severity of the asymmetry depends upon the phyllotactic direction of the plant as well. This suggests that the intrinsic asymmetry imparted by resupination conflicts specifically with one phyllotactic direction but not the other. We then analyze symmetrical sources of shape variance and overall leaf size, and show that leaves arising from plants with different phyllotactic directions differ in their (1) symmetrical shape and (2) size. We propose that the morphological forces shaping the asymmetry of *A. psittacina* leaves – resupination and phyllotaxis – interact with each other and either promote or hinder leaf development in a phyllotaxis-dependent manner, creating leaves of different shapes and sizes from plants with opposing phyllotactic directions. The genetic and evolutionary implications of the conflict between phyllotactic chirality and overall morphological form are discussed.

## Results

### Intrinsic asymmetry in *A. psittacina* leaves likely results from invariant resupination

We hypothesized that the resupination present in *A. psittacina* might impart asymmetry in its leaves. Because the leaves invariantly turn in the same direction, regardless of the phyllotactic direction (Figure 1), such an asymmetry would be present in all leaves. To measure such an asymmetry, we photographed both sides of leaves, the ab.-top and ad.-bottom. The two images of a leaf, from its opposite sides, are mirror images of each other. Therefore, if the asymmetries in leaves are random, they will, on average, cancel each other out, and the averaged images of the ab.-top and ad.-bottom sides of the leaf will be more or less identical. However, if an asymmetric bias exists in all leaves, then we would expect that the ab.-top and adaixal-bottom sides of the leaf would not be identical. In fact, they would be mirror images of each other, and their incongruence would be reflective of the overall asymmetry present in leaves.

**Figure 2:**
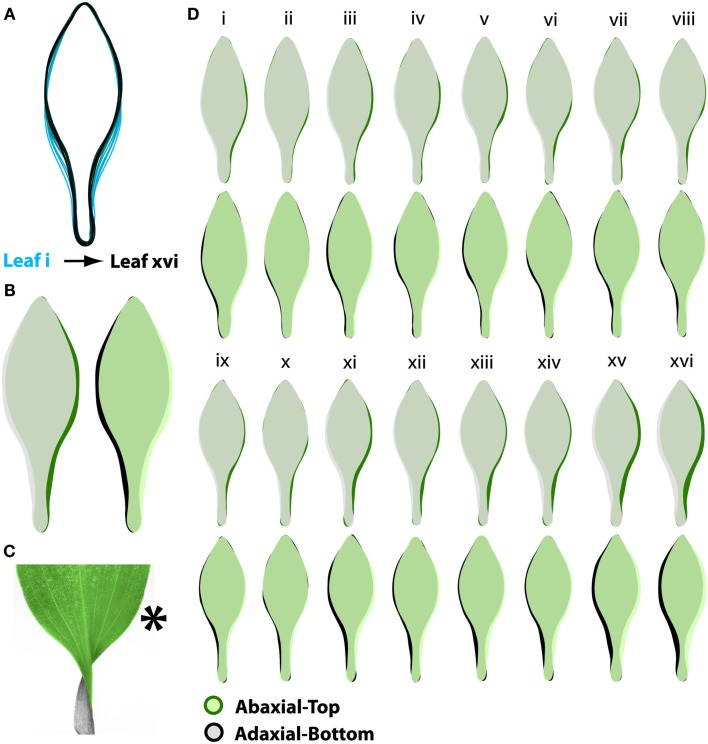
***A. psittacina* leaves exhibit intrinsic asymmetry, likely attributable to the invariant direction of resupination**. **(A)** To demonstrate changes in leaf shape that occur across the leaf series, mean leaf outlines beginning with the first sampled leaf closest to the apex (i) and the last sampled leaf on the shoot (xvi) are superimposed. Note that leaves sampled on nodes further from the apex attain a relatively more distinct petiole and shift the distribution of their blade outgrowth distally compared to leaves nearer the apex. **(B)** In order to detect intrinsic asymmetry in leaves, we photographed both sides of a leaf. Shown are superimposed outlines of abaxial-top (green) and adaxial-bottom (black) averaged images. On the left, adaxial-bottom is placed over abaxial-top, and on the right *vice versa*. Note that there is more laminar outgrowth on the right side of the abaxial-top images, suggesting intrinsic asymmetry. **(C)** To place in perspective the asymmetries shown in **(B)**, a resupinate leaf is shown false-colored green and gray on its abaxial-top and adaxial-bottom sides, respectively. The asterisk denotes the side of the leaf on which excess blade outgrowth is observed. **(D)** Outlines of abaxial-top and adaxial-bottom images at each position in the leaf series. Note the consistency (albeit less severe) of the asymmetries at each node compared to **(B)**, in which leaves at all nodes are averaged together.

To answer these questions, we imaged >2,300 leaves from >240 shoots of *A. psittacina*. Each leaf was flattened, so that its resupinate twist was no longer present, and the ab.-top and ad.-bottom sides of the leaves were scanned. Leaves were organized by their shoot position (“i” denotes the node closest to the apex, and we analyze data up to node “xvi” away from the apex). We began by looking at averaged outlines of the leaves. To understand the shape changes that occur through the leaf series, we superimposed the averaged outlines of leaves by the node from which they originate. Proceeding from the apex downwards, a trend by which the petiole region becomes more distinct and the distribution of laminar outgrowth becomes shifted distally is evident (Figure 2A). It should be noted that the leaf series is confounded for heteroblastic changes in leaf shape and the age of leaves, and that the influence of these two factors on the shape changes in leaves has yet to be resolved.

If one then superimposes the outlines of the ab.-top and ad.-bottom sides of leaves, a clear indication of asymmetry is obvious. Because the asymmetry represents a small portion of the overall leaf area, we superimposed leaf outlines in each combination – ab.-top over ad.-bot., and ad.-bot. over ab.-top. Looking at the averaged outlines from each side of the leaf (Figure 2B), it is clear that there is preponderance of laminar outgrowth on the right side of the ab.-top outlines (green) relative to the ad.-bottom side (black). The converse is true when analyzing the ad.-bottom side of the leaf relative to the ab.-top. Where do these asymmetries lie with respect to a resupinate leaf? Looking at a resupinate leaf (Figure 2C), positioned such that the blade is the ab.-top surface and the base of the petiole is the ad.-bottom surface, the results predict an excess of blade outgrowth on the right side of the blade (from the perspective of the ab.-top surface as shown in Figure 2C). This asymmetry, evident in averaged leaves across the leaf series, can be observed at individual nodes as well, albeit with more noise in the averaged outlines because of the fewer samples represented at each position (Figure 2D).

The asymmetries we observe, which manifest as biases in the outlines of ab.-top images relative to ad.-bottom, suggest that the leaves of *A. psittacina* possess intrinsic asymmetry. Such an asymmetry is consistent with the direction of resupination in *A. psittacina*, which is invariant in all leaves, regardless of phyllotactic direction.

### Quantification of the sources of asymmetry in *A. psittacina*

**Figure 3:**
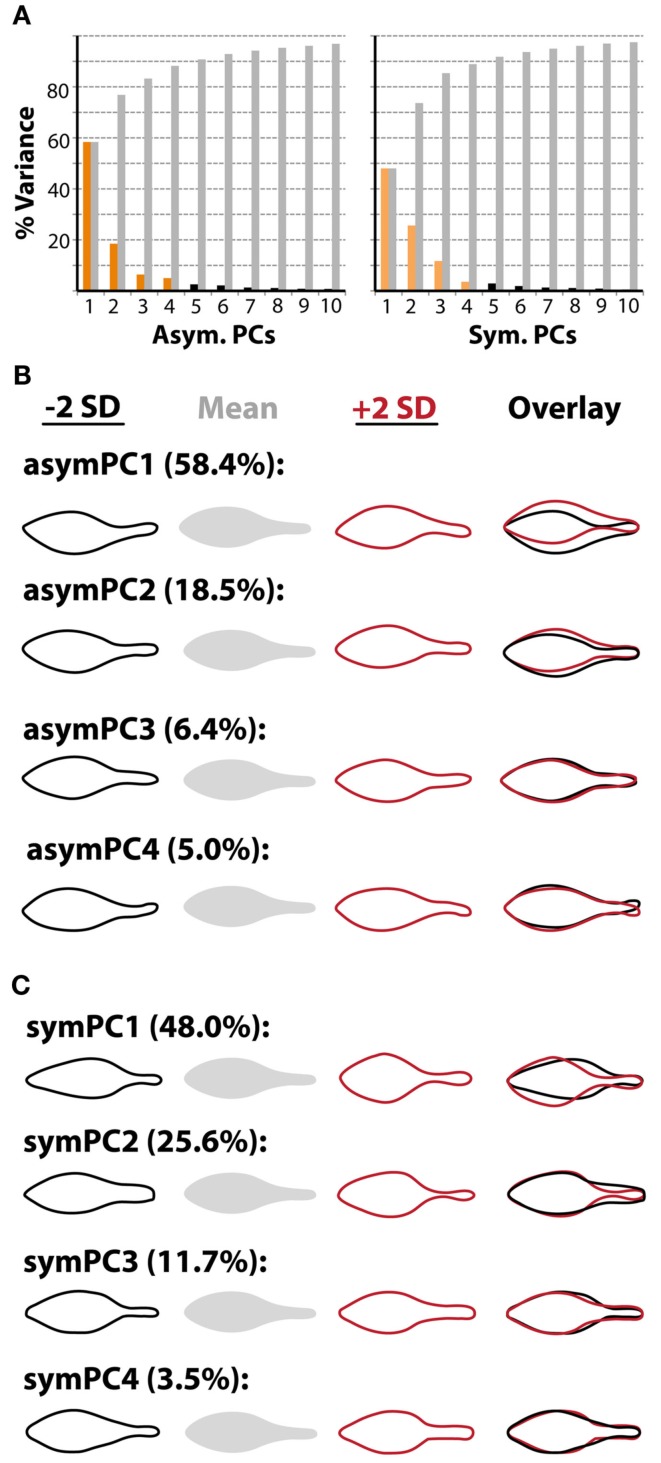
**Principal components describing symmetrical and asymmetrical shape variance**. **(A)** Both abaxial-top and adaxial-bottom images were used in an Elliptical Fourier Descriptor (EFD) analysis to examine asymmetric shape variance. Abaxial-top images only were used in an analysis of symmetric shape variance, as mirror images were not needed to analyze asymmetry. Shown is the percent variance in asymmetric (left) and symmetric (right) analyses explained by each PC. Note that the amount of variance described by the first PCs (asymPC1: 58.4%, symPC1: 48.0%) is relatively high, and that collectively the first four PCs describe a large amount of the overall variance (asymPCs1–4: 88.3%, symPCs1–4: 88.8%). **(B)** Leaflet outlines representing ±2 standard deviations along asymmetric principal component axes. Particular attention is given to asymPC1 in this study, which explains asymmetric variance relating to overall bending of the leaf. **(C)** Leaflet outlines representing ±2 standard deviations along symmetric principal component axes. Particular attention is given to symPC1, which, like the other symPCs, describes symmetric shape variance relating to the distinctness of the petiole and distribution of laminar outgrowth along the proximal-distal axis.

To not only quantify the asymmetries we observe, but to determine which factors modulate them, we conducted an Elliptical Fourier Descriptor (EFD) analysis of leaf outlines (Iwata et al., 1998; Iwata and Ukai, 2002). A powerful feature of EFD is the ability to separate asymmetric sources of shape variance from symmetric, which is important for the question of hand. Compared to a previous study (Chitwood et al., 2012b), large amounts of shape variance (∼50%) are represented by the first principal components (PCs) describing symmetric and asymmetric variance, likely because a within genotype comparison is being made (Figure 3A). We consider the first four asymmetric and symmetric PCs, which describe in total 88.3 and 88.8% of the respective shape variances. The first asymmetric PC, describing ∼58% of all asymmetry, describes shape variance relating to the bending of the petiole and blade, and later we focus more on this particular PC (Figure 3B). The symmetric PCs by and large explain variance relating to the distinctness of the petiole and the distribution of laminar outgrowth along the proximal-distal axis of the leaf (Figure 3C).

**Figure 4:**
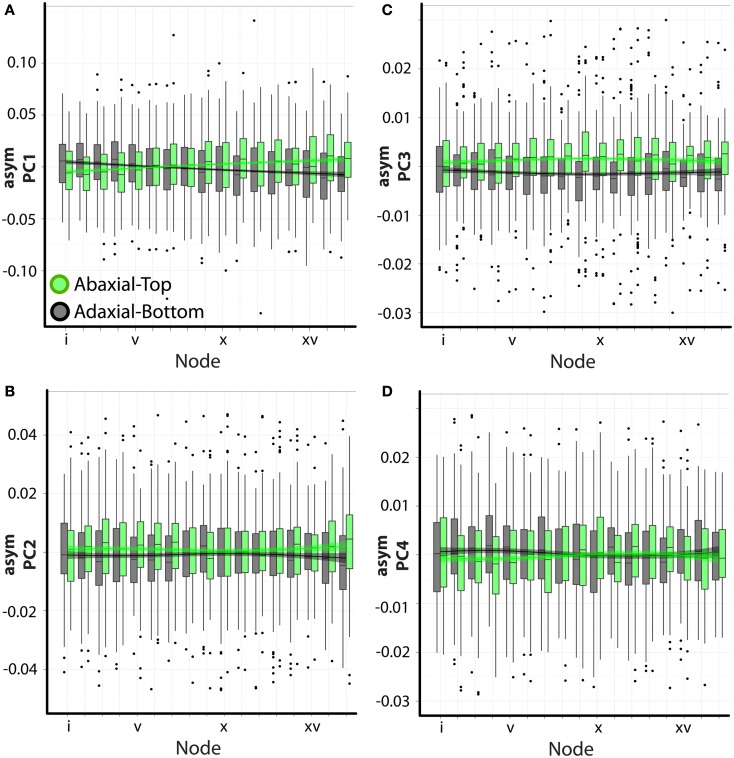
**Visualization of asymmetric ANOVA results**. **(A–D)** Shown are barplots with superimposed loess regression lines (with standard error bands). asymPC1–4 [**(A–D)**, respectively] values are separated by abaxial-top (green) and adaxial-bottom (gray) sides. Node position is denoted on the *x*-axis (lower values correspond to positions closer to the apex). Note that the significant differences in PC values between abaxial-top and adaxial-bottom outlines for PCs 1–4, signifying intrinsic asymmetry, are easily observed. Additionally, various types of interactions between the abaxial-top/adaxial-bottom factor with node position are present, denoting that intrinsic asymmetry changes through the leaf series.

We used an ANOVA model to determine the various factors significantly correlated with the asymmetries we observe. Again, the analysis of asymmetric variance relies on images representing both the ab.-top and ad.-bottom sides of the leaf. Significant differences between ab.-top and ad.-bot. leaves signifies an intrinsic asymmetry in leaves. Both position in the leaf series and phyllotactic direction do not significantly explain the asymmetric variance represented by any PC (Table 1). This is expected: at each node position, and for each phyllotactic direction, the ab.-top and ad.-bot. images “cancel” their asymmetries, as they are mirror images of each other. However, if a comparison of the asymmetry in ab.-top and ad.-bot. images is made against each other, all PCs show significant differences in their values (Table 1). Although the data is highly variable, this trend is easily visualized (Figure 4). The significant differences in asymmetric PC values between ab.-top and ad.- bot. images of the leaf demonstrate that the leaves of *A. psittacina* exhibit an intrinsic asymmetry (as shown in Figures 2B–D) that we believe is likely caused by the invariant direction of resupination found in this species. This intrinsic asymmetry changes in a way dependent on the position of the leaf in the leaf series, as demonstrated by the significant interaction term between node position and adaxial vs. abaxial images for PCs 1–4 (Table 1; Figure 4). To examine a single PC in detail, asymPC1, which explains ∼58% of all asymmetric shape variance, shows an interesting property, in which the asymmetries exhibited by the ab.-top and ad.-bot. sides of the leaf invert early in the leaf series (Figure 4A). As previously mentioned, resupination occurs relatively late after leaf initiation, and the first leaves we sampled from each shoot had yet to completely invert (Figure 1A). As a PC explaining a majority of leaf asymmetry, asymPC1 behaves in a manner consistent with the developmental onset of resupination.

**Table 1:** **ANOVA results for leaf area and PCs explaining asymmetric variance**.

Factor	asymPC1	asymPC2	asymPC3	asymPC4
Position	ns	ns	ns	ns
Phyllotaxy	ns	ns	ns	ns
AdAb	2.9 × 10^−4^	1.7 × 10^−9^	3.5 × 10^−11^	4.1 × 10^−3^
Position:phyllotaxy	ns	ns	ns	ns
Position:AdAb	2.9 × 10^−15^	2.7 × 10^−2^	9.2 × 10^−7^	1.0 × 10^−8^
Phyllotaxy:AdAb	1.4 × 10^−5^	ns	ns	ns
Position:phyllotaxy:AdAb	6.4 × 10^−4^	4.0 × 10^−5^	3.5 × 10^−6^	2.1 × 10^−4^

*Significance values for factors explaining the variance in asymPCs 1–4. In this analysis, both abaxial-top and adaxial-bottom images were considered to detect intrinsic leaf asymmetries. Because of this, it is predicted that node position (“Position”) and phyllotactic direction (“Phyllotaxy”) are not significant (n.s.), as ab.-top and ad.-bot. images “cancel-out” their asymmetries. However, the significant difference for the ab.-top/ad.-bottom factor (AdAb) shows that *A. psittacina* leaves posses intrinsic asymmetry. Additionally, this asymmetry is significantly modulated by node position (“Position:AdAb”) and phyllotaxy (“Phyllotaxy:AdAb”)*.

**Figure 5:**
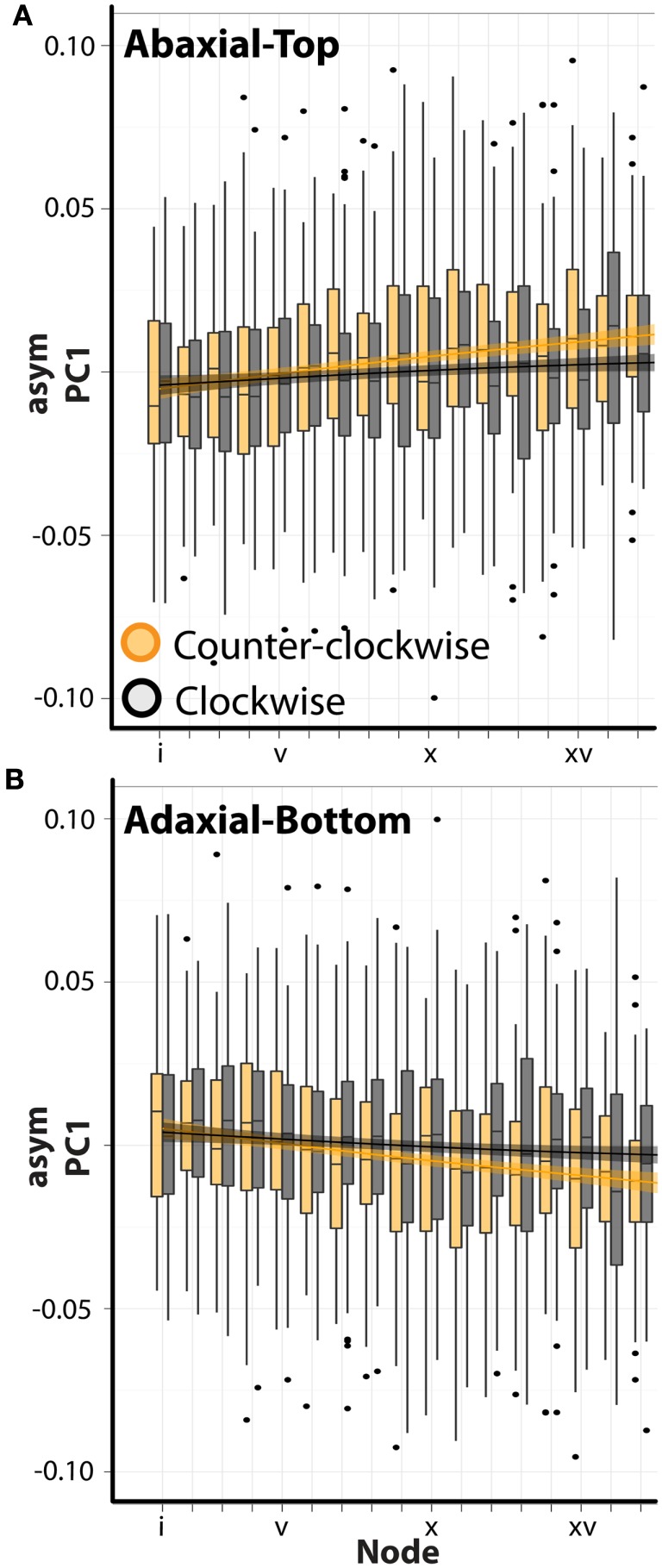
**Leaves arising from plants with opposing phyllotactic directions possess different degrees of asymmetry**. **(A)** asymPC1 values for abaxial-top images separated by leaves arising from clockwise (gray, CL) and counter-clockwise (orange, CC) plants. Note the more extreme asymmetry exhibited by leaves from CC plants. *x*-axis shows node position (lower values closer to the apex). Superimposed upon barplots are loess regression lines with standard error bands. **(B)** The same as shown in **(A)**, except for adaxial-bottom images. Note that the values are mirror images of those shown in **(A)**, as the adaxial-bottom image of a leaf is the mirror of the abaxial-top image. Again, leaves arising from counter-clockwise plants exhibit more extreme asymmetry.

Interestingly, there is a significant interaction between phyllotactic direction and ab.-top vs. ad.-bot. images with respect to asymPC1 (Table 1). There is even a significant triple interaction for PCs 1–4. This suggests that the changes in intrinsic leaf asymmetry (which we presume are reflective of resupination) are dependent upon phyllotactic direction. These relationships can be visualized for asymPC1, which explains a majority of the asymmetric shape variance (Figure 5). Separating out the trends between clockwise (black, CL) and counter-clockwise (orange, CC) phyllotactic directions for the ab.-top (Figure 5A) vs. ad.-bottom (Figure 5B) images of leaves, it is obvious that leaves arising from CC plants are intrinsically more asymmetric. That is, whereas leaves from CL plants exhibit asymPC1 values closer to zero throughout the series, leaves from CC plants proceed to higher, more extreme asymPC1 values through the leaf series in ab.-top images (Figure 5A), and lower asymPC1 values through the leaf series in ad.-bottom images (Figure 5B; reflecting the overall trends between ab.-top vs. ad.-bot. images shown in Figure 4A).

It is obvious that the intrinsic asymmetry of leaves in *A. psittacina* is dependent upon phyllotactic direction and node position. One possible explanation is that resupination, which is responsible for the intrinsic asymmetry, is a marker of the developmental state of the leaf, and that CL leaves are not necessarily less asymmetric than those leaves arising from CC plants, but just developmentally delayed.

### Developmental delay in the shape and size of leaves conflicted by intrinsic asymmetry and phyllotaxy

Previous work has demonstrated that in superficially bilaterally symmetric leaves asymmetry is phyllotactically dependent, and that leaves arising from plants of opposite phyllotactic directions are mirror images of each other (Chitwood et al., 2012a). Our results suggest that *A. psittacina* leaves from equivalent nodes of plants with opposite phyllotactic directions are not simply mirror images: leaves from CC plants are intrinsically more asymmetric (Figure 5). This is not to say that leaves at nodes further from the apex in CL plants cannot achieve a similar degree of asymmetry, which is suggestive of a developmental delay. But if indeed leaves arising from CL plants are developmentally delayed, a number of corollaries ensue: at equivalent nodes, CL leaves should be (1) smaller and (2) have more juvenile shapes relative to comparable CC leaves.

**Figure 6:**
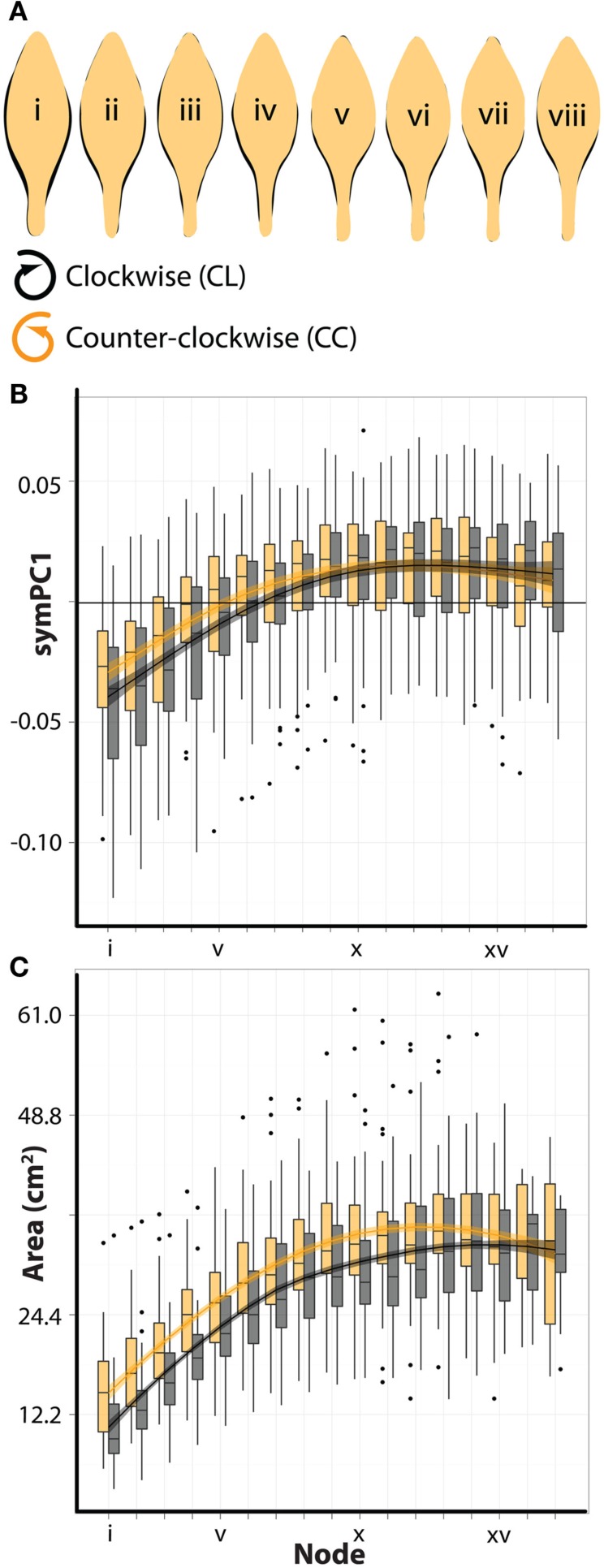
**Counter-clockwise plants produce larger leaves with more developed shape characteristics**. **(A)** Shown are juxtaposed mean outlines of abaxial-top leaves arising from clockwise (black, CL) and counter-clockwise (orange, CC) plants for the first eight nodes from the apex. Note that leaves arising from CC plants have more defined petioles and the distribution of their laminar outgrowth is shifted distally compared to leaves from CL plants – characteristics typical of more developed leaves found farther in the series (see Figure 2A). **(B)** symPC1 values for abaxial-top leaves separated by leaves arising from clockwise (black, CL) and counter-clockwise (orange, CC) plants. Superimposed upon barplots are loess regression lines with standard error bands. *x*-axis shows node position (lower values closer to the apex). CL symPC1 values are shifted right relative to CC values across the leaf series, suggesting a developmental delay. **(C)** Similar to (B), but showing the overall blade area (cm^2^) of leaves. Note again that CL leaves are shifted right relative to CC leaves, suggesting a developmental delay.

To analyze the developmental characteristics of leaves arising from plants of opposite phyllotactic directions, we examined symmetrical shape variance (Figure 3C) and leaf area. ANOVA results suggest that symmetrical shape varies significantly for all PCs by node position (Table 2). This is not surprising, as most the symPCs explain shape variance relating to the distinctness of the petiole and the distribution of laminar outgrowth along the proximal-distal axis of the leaf (Figure 3C), which upon an examination of superimposed mean outlines at different nodes (Figure 2A), varies across the leaf series. Remarkably, symPC1 (which explains 48% of all symmetrical shape variance) varies significantly by phyllotactic direction as well, demonstrating that leaves arising from plants with opposing phyllotactic directions have different shapes (Table 2). Additionally, symPC2 possesses a significant interaction term between phyllotactic direction and node position. A closer inspection of how leaves are differently shaped based on node position and phyllotactic direction is reminiscent of the changes through the leaf series (Figure 2A): leaves from CC plants have more distinct petioles and more distally distributed blade relative to CL leaves, characteristics typical of leaves farther in the leaf series (Figure 6A). These shape differences between CL and CC leaves can be visualized as a shift of CL PC values right relative to CC values, toward nodes farther away from the apex (Figure 6B).

**Table 2:** **ANOVA results for leaf area and PCs explaining symmetric variance**.

Factor	symPC1	symPC2	symPC3	symPC4	Area
Position	<2.2 × 10^−16^	<2.2 × 10^−16^	9.5 × 10^−11^	8.4 × 10^−7^	< 2.2 × 10^−16^
Phyllotaxy	6.3 × 10^−3^	ns	ns	ns	1.46 × 10^−11^
Position:phyllotaxy	ns	3.8 × 10^−2^	ns	ns	ns

*Significance values for factors explaining the variance in symPCs 1–4 and leaf area. In this analysis, only abaxial-top images were considered. Symmetric shape and leaf area change significantly by node position (“Position”) and phyllotactic direction (“Phyllotaxy”). There is also a significant interaction effect (“Position:Phyllotaxy”) detected for a symmetrical shape PC*.

The developmental delay of heteroblastic changes in shape between leaves from plants of different phyllotactic directions is strikingly observed in leaf area as well (Table 2; Figure 6C): leaves from CL plants at equivalent node positions are smaller than CC leaves. That differences in the shape and size of leaves between CC and CL plants represent developmental delays rather than intrinsic differences is exemplified by the fact that for each, CL leaves eventually “catch up” and attain equivalent CC values in mature leaves farther from the apex (Figures 6B,C).

**Figure 7:**
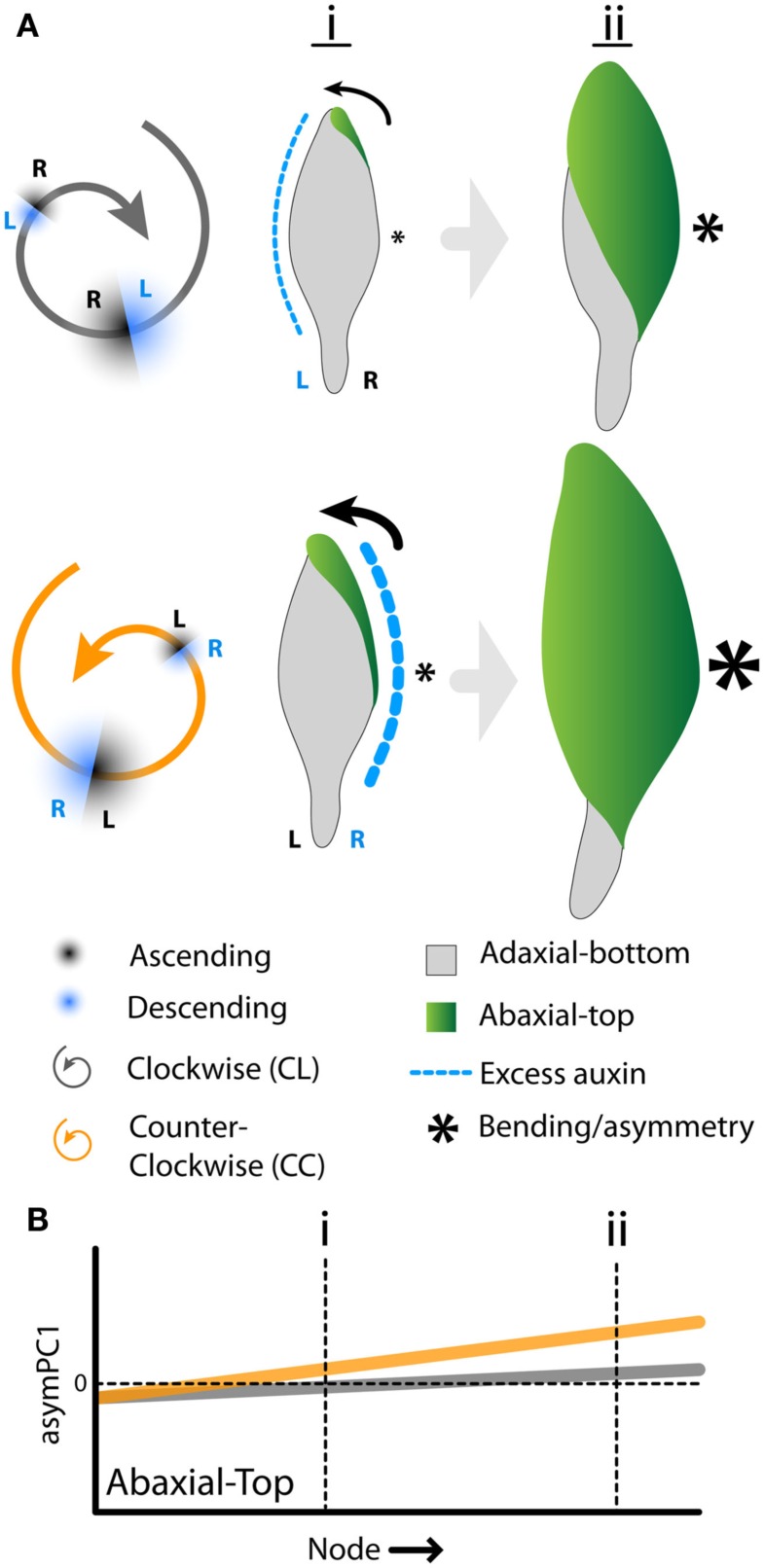
**Interaction between phyllotactic-dependent asymmetry and resupination**. **(A)** Model of how phylotactic dependent asymmetry either facilitates or hinders resupination. In clockwise phyllotaxis (black) the descending side of the leaf (toward the older neighbor) is the left side. Auxin is biased toward the left side of the leaf (dotted blue line) where it presumably creates excess blade outgrowth and a distal shifting of features. Resupination, however, begins on the right side of the leaf with the folding over of blade (arrow). In counter-clockwise phyllotaxis (CC, orange), the auxin bias (blue dotted line), the excess blade outgrowth it creates, and resupination (arrow) all occur together on the right side of the leaf. Presumably the excess blade outgrowth generated by the auxin bias facilities resupination. As resupination is intimately tied to the development of leaves, this allows CC to progress through their developmental program faster, generating larger leaves at early (i) and later (ii) nodes relative to CL leaves. Additionally, the arcing of leaves away from the left (asterisk size) induced by resupination proceeds to a more extreme state in CC phyllotaxis. **(B)** Diagrammatic representation of the data shown in Figure 5 for abaxial-top images. The positive slope of asymPC1 values in leaves as one progresses away from the apex indicates an intrinsic asymmetry in these leaves. CC leaves are more asymmetric than CL leaves. However, in light of the eventual convergence in shape (Figure 6B) and area (Figure 6C) between CL and CC leaves suggestive of developmental delay, CL leaves likely eventually attain CC asymmetric values, albeit at nodes farther from the apex. This interpretation suggests that intrinsic asymmetry results from the developmental progression of leaves and the resupination process.

The developmental delay of CL leaves results from an interaction between phyllotactic direction and intrinsic leaf asymmetry arising from resupination. Mechanistically, how might these two processes create the differences we observe between CC and CL plants? It is known from previous work, using modeling and molecular reporters, that there is a bias in auxin distribution toward the descending side of a leaf primordium, i.e., the side closer to the older neighboring leaf (Chitwood et al., 2012a). In tomato and *Arabidopsis*, the excess auxin causes increased laminar outgrowth and the shifting of features, such as leaflets and lobes, toward the distal tip of the leaf. In *A. psittacina*, resupination begins by the folding over of the ab.-top surface on the right side of the leaf (Figure 1A). This occurs whether or not the phyllotactic spiral is CL or CC (Figure 7A). We propose that, as in other organisms (Korn, 2006; Chitwood et al., 2012a), *A. psittacina* leaves experience excess blade outgrowth on their descending side. In CC plants, the descending side (right) is congruent with the outgrowth required to initiate the twisting of the leaf, facilitating the process of resupination. However, in CL plants, the descending side (left) is opposite the side of the leaf that folds over, delaying the twisting of the leaf. Resupination is a dynamic process that facilitates the developmental outcome of *Alstroemeria* leaves. That this process is facilitated by the circumstances of phyllotaxy in CC plants ultimately creates larger leaves (Table 2; Figure 6C) that attain more mature shapes relative to their CL counterparts (Table 2; Figures 6A,B) by accelerating their development. That it is resupination that is a key element in this process is reflected in the fact that CC leaves are more asymmetric than CL leaves (Figure 5). By proceeding farther through the resupination process, they attain more quickly the characteristic asymmetry imparted by it: a bending of the leaf away from its left side as viewed from the ab.-top surface (Figures 2B–D, 3B, 5, and 7B).

## Discussion

Handedness has received considerable attention in animals. Conserved across the bilateria is the asymmetric expression of *Nodal* and *Pitx* (Levin et al., 1995; Collignon et al., 1996; Shapiro et al., 2004; Grande and Patel, 2009). The patterning of these factors depends on phylogenetic context, and although still debated, it is thought to involve cytoskeletal elements, whether through the directional beating of cilia during gastrulation, inherent chirality of the cytoskeletal system, or segregation of differentially imprinted chromatids (Afzelius, 1976; Nonaka et al., 1998; Okada et al., 1999; Vandenberg and Levin, 2010). Handedness, an invariant condition of particular chiral state, is distinct from random generation of asymmetry, in which enantiomorphs are assigned randomly in a population without a genetic basis (Brown and Wolpert, 1990). Such a condition can be observed in the ∼50:50 ratio of *situs inversus* and *situs solitus* mice harboring the *iv* (*situs inversus*) mutation, in which handedness is not dependent upon the genotype of the parent. However, when handedness is genetically specified (e.g., the maternal inheritance of the sinistral, *l*, and dextral*, L*, condition in pulmonate snails; Sturtevant, 1923), segregation of enantiomorphs can be selected upon. Examples of adaptive, genetically specified asymmetries include the frequency-dependent selection on the handedness of mouth opening in scale-eating cichlid fishes (Hori, 1993) and the number of teeth on each side of the jaw in snail-eating snakes (Hoso et al., 2007). However, the term enantiomorph can be misleading, as the phenotypes of oppositely chiral individuals may not be perfect mirror images of each other (Gould et al., 1985; Johnson, 1987), suggesting that the genetic determinants of left-right asymmetry may be multifaceted, and that other developmental constraints may modulate asymmetry in a chiral-dependent manner.

Like animals, in plants the most extensively described asymmetry (1) concerns cytoskeletal components and (2) involves genetically specified handedness of a single enantiomorph. A variety of microtubule mutants can confer extreme handedness, creating torsions from the molecular level, to cells, to whole plant morphology, even approximating resupinate leaves (Hashimoto, 2002; Thitamadee et al., 2002). The invariant direction of twining in various taxa of vines has been hypothesized to potentially result from genetic causes modulating the handedness of microtubules (Hashimoto, 2002). The resupination present in *Alstroemeria* and *Bomarea*, which we show in *A. psittacina* always occurs in a single direction (Figure 1) and confers asymmetry of a particular handedness to all leaves (Figures 2–4; Table 1), may similarly be under such genetic controls. Considering that some species of *Bomarea* are climbing vines (Hill, 1939; Hofreiter and Lyshede, 2006), it will be interesting to see if twining orientation can be correlated with resupination and to investigate the genetic properties of the microtubules variants harbored in Alstroemeriaceae. Indeed, resupination may be an adaptation to the versatility of *Bomarea* vines to climb upwards, downwards, and grow horizontally, always orienting the ab.-top surface of the leaf toward light, even if this leads to untwisting or double twisting (Hill, 1939).

*Alstroemeria psittacina*, like many other Angiosperms, exhibits spiral phyllotaxy, and as we have previously shown (Chitwood et al., 2012a), leaves often exhibit asymmetry in a phyllotactic-dependent manner. This is true of *A. psittacina* as well, in that the intrinsic asymmetry imparted by resupination is modulated in a phyllotactic-dependent manner (Table 1), such that leaves arising from CC plants are more asymmetric than CL leaves (Figure 5). Phyllotactic-dependent asymmetry is unlike most asymmetry in animals; it is randomly generated, and not dependent upon the chirality of the parent. What makes *A. psittacina* unique compared to other asymmetry studies is the coexistence of genetically specified handedness (resupination) and other randomly generated asymmetries (phyllotaxis), and how the interaction between these two factors extends beyond asymmetry to affect the overall development of the leaf. Leaves arising from CC plants are not just more asymmetric, but are larger and have symmetrical shape characteristics of mature leaves (Figure 6). Analyzing these morphological attributes by node, it becomes apparent that CL leaves are developmentally delayed, as eventually they attain phenotypes equivalent to CC plants, albeit at more basal node positions (Figure 6).

Stephen Jay Gould, and others, commented on rare sinistral forms of snails in otherwise dextral populations. Was it that the sinistral form was selected against *per se*? In fact, the sinistral forms were not mirror images of the dextral form, and the contributions of other features in addition to the sinistral form must be evaluated when considering the fitness of sinistral snails in this species (Gould et al., 1985; Johnson, 1987). The leaves of *A. psittacina* are similar: leaves from CL and CC plants are not equivalent in form or developmental rate because of the interaction between phyllotaxis and resupination (Figures 6 and 7). However, there is a key difference between the snails described by Gould et al. (1985) and Johnson (1987) vs. *A. psittacina* in that there is no genetic basis for phyllotactic direction. Even if plants of a particular phyllotactic direction are less fit, there is no genetic basis for selection to act upon. If phyllotactic direction had a genetic basis, one might imagine that the holistic phenotype characteristic of *Alstroemeria* of one phyllotactic direction or another could be acted upon by directional selection, yielding a species not unlike some snails, with a predominate enantiomorph. The stochastic origin of phyllotactic direction in plants makes phyllotactic-dependent asymmetry unique compared to the asymmetric forms commonly found in animals, which are typically vastly biased to a particular enantiomorph. It will be interesting to explore asymmetry further in those species that exhibit handedness in their phyllotaxy, as has been reported for *Calathea* (Korn, 2006). As phyllotactic-dependent asymmetry has been established (Chitwood et al., 2012a), as well as its interactions with other sources of asymmetry, the next question to answer is why phyllotaxy so rarely exhibits fixed handedness.

## Materials and Methods

### Plant materials

*Alstroemeria psittacina* (accession number A92.0412) shoots were obtained from the U.C. Davis Arboretum. Material was collected from a population located in a riparian context, slightly shaded by trees, along Putah Creek in Davis, CA. Material was collected over a ∼2 week period near the end of February and continuing into March 2012.The *A. psittacina* population during this time exhibited prolific vegetative growth and had yet to transition into flowering. Although the population exhibited no bias in phyllotactic direction, equal numbers of each phyllotactic direction were collected during each outing.

### Data collection

Whole shoots were collected and brought back to lab in zip lock bags. The most mature leaves farthest from the apex, excluding those that had degraded and senesced beyond a usable context, were included in the analysis. Leaves were cataloged by the node from which they emerged, beginning with the first node used closest to the apex as (i). The first leaf that was large enough to be manipulated (i.e., had uncoiled and proceeded through its vernation enough that it could be flattened), usually greater than 2–3 cm in length, was used. The first few leaves tended to have begun their resupinate twisting, but had yet to completely twist. The second or third leaf used for each shoot, however, had usually completely twisted 180°. For each shoot, leaves were flattened out and adhered to white construction paper using repositionable adhesive spray (Scotch). With the ab.-top side of the leaf facing upwards, leaves were then scanned (Epson, Perfection V300). Leaves were then removed, turned over, and their ad.-bottom side was scanned. In total, 248 shoots, with equal numbers of each phyllotactic direction, were analyzed. 4,656 images of ab.-top and ad.-bottom outlines, derived from 2,328 leaves, were analyzed. On average, approximately nine leaves per shoot were analyzed.

Leaf outlines were then extracted as binary images using ImageJ (Abramoff et al., 2004), their overall area recorded in the process, and saved as appropriately named, individual files.

### Elliptical Fourier Descriptors

The analysis of leaf shape was conducted using EFDs followed by PCA using the program SHAPE (Iwata and Ukai, 2002). Object contours were extracted as chain-code. Chain-code was subsequently used to calculate normalized EFDs. Normalization was based upon manual orientation with respect to the proximal-distal axis of the leaf. Principal component analysis was performed on the EFDs resulting from the first 20 harmonics of Fourier coefficients. For the analysis of symmetrical shape, *a* and *d* coefficients were analyzed, while for analysis of asymmetrical shape, *b* and *c* coefficients were analyzed (Iwata et al., 1998). Coefficients of EFDs were calculated at −2 and +2 standard deviations for each principal component and the respective contour shapes reconstructed from an inverse Fourier transformation. PCs were then analyzed for statistical differences between various factors.

### Statistical analysis and visualization

All statistical analysis were performed in R (R Development Core Team, 2011). ANOVA modeling was performed using a backward model selection process comparing models differing by a single term. *p*-values for significant terms represent final model results. Non-significance of terms was verified by comparing models with added terms using a forward model selection process.

Results were visualized using the ggplot2 package in R (Wickham, 2009).

## Conflict of Interest Statement

The authors declare that the research was conducted in the absence of any commercial or financial relationships that could be construed as a potential conflict of interest.

## Supplementary Material

The Supplementary Material for this article can be found online at http://www.frontiersin.org/Plant_Evolution_and_Development/10.3389/fpls.2012.00182/abstract
